# Significance of the progesterone receptor and epidermal growth factor receptor, but not the estrogen receptor, in chemically induced lung carcinogenesis in female A/J mice

**DOI:** 10.3892/ol.2014.2559

**Published:** 2014-09-24

**Authors:** SOSUKE KISHI, MASANAO YOKOHIRA, KEIKO YAMAKAWA, KOUSUKE SAOO, KATSUMI IMAIDA

**Affiliations:** 1 Department of Pathology and Host-Defense, Faculty of Medicine, Kagawa University, Kagawa 761-0793, Japan; 2 Department of Diagnostic Pathology, Tomakomai City Hospital, Hokkaido 053-0034, Japan

**Keywords:** lung carcinogenesis, progesterone receptor, estrogen receptor, epidermal growth factor receptor, non-small cell lung cancer

## Abstract

In the present study, the expression levels of female hormone receptors, estrogen receptor (ER) and progesterone receptor (PR) and the epidermal growth factor receptor, (EGFR), as well as proliferating cell nuclear antigen (PCNA) were examined in lung tumors that were induced by 4-(methylnitrosamino)-1-(3-pyridyl)-1-butanone (NNK) in female A/J mice. Each seven-week-old mouse was administered with 2 mg NNK via intraperitoneal injection and the mice were subsequently euthanized at week 52. Lung tumors, including adenomas, carcinomas in adenomas and adenocarcinomas, were obtained and analyzed by immunohistochemistry for the expression levels of the receptors, ER, PR and EGFR, and PCNA. The results were as follows: i) In mouse lung adenomas, a significant correlation was identified between the size of the tumor and PCNA expression, although not with the expression of the receptors (ER, PR and EGFR); ii) in the carcinoma components of the carcinomas in adenomas, the size of the tumor and PCNA expression were correlated, while EGFR expression demonstrated a significant correlation with PR expression; iii) in adenocarcinomas, the tumor size significantly correlated with PCNA, EGFR and PR expression; and iv) EGFR and PR expression was identified to be significantly correlated in adenocarcinomas, and to a certain extent in the carcinoma components of the carcinomas in adenomas, although not in the adenomas. Notably, ER expression was not associated with tumor growth or the other factors, particularly EGFR expression, and no significant differences were identified between the three types of lesion. These results indicate that PR, like EGFR, may be significant in lung carcinogenesis.

## Introduction

Lung cancer is the most common cause of cancer-associated mortality in males and females worldwide (1). Non-small cell lung cancer (NSCLC) is the predominant type of lung cancer, including the major histologic types, such as adeno-, squamous cell and large cell carcinoma. In addition, advanced NSCLC is the most common cause of cancer-associated mortality in males and females in the United States (2). Furthermore, a large number of individuals globally suffer from NSCLC, thus, novel treatment and prevention approaches for this disease are urgently required.

Despite a decline in the smoking population, the incidence of NSCLC, particularly the adenocarcinoma subtype, continues to increase (3,4). Adenocarcinoma is the most frequent histologic type of NSCLC in non-smokers and young patients (4,5). This indicates that etiologic factors other than tobacco may be involved in the development and progression of NSCLC.

Numerous studies have reported that female sex hormones, particularly endogenous and exogenous estrogens, may contribute to NSCLC in females (6–9), and estrogen receptors (ERs) are expressed in NSCLC tissue and may be associated with neoplasia (6–11). Nuclear and extranuclear ERs appear to act concomitantly to promote cell growth (12,13).

In the normal lung tissue of humans and rodents, ERs perform important physiological functions to promote proliferation, including those that are required for alveolar formation in the development, alveolar regeneration and maintenance of the pulmonary diffusion capacity (14,15).

The progesterone receptor (PR) mediates the effect of progestins and it has been reported that the progression of spontaneous lung tumors was reduced by treatment with antiprogestin (Mifepristone, also termed RU-486) in mice (16). However, the association between the intensity of PR expression and the biological characteristics of lung cancer tissue remains unclear. Furthermore, PR expression in human lung tumors varies markedly, from a high expression frequency of 39–63% (17–19) to marginal (22–35%) or no expression (20,21).

It has previously been reported that the subset of patients with NSCLC, predominantly those with adenocarcinomas, possess a specific activating mutation in the epidermal growth factor receptor (EGFR) gene, which correlates with marked clinical responsiveness to the EGFR tyrosine kinase inhibitors, gefitinib or erlotinib (22–24). This was subsequently identified by studies, which consistently demonstrated that mutations in the tyrosine kinase domain of EGFR are more commonly found among females, never-smokers, Asian individuals and those exhibiting adenocarcinomas (25–30). However, despite an almost universal presence of EGFR in NSCLC tumors, the therapeutic inhibition of EGFR by gefitinib and erlotinib results in significant tumor regression in only 10–20% of patients, again indicating the variation in the molecular pathogenesis of the different types of lung cancer.

The primary causes of NSCLC, not including tobacco smoking, remain unclear. Although sex hormones have been implicated as a cause of lung cancer in non-smoking females there are numerous additional unidentified mechanisms. The aim of the present study was to elucidate the pathognomonic factors that promote the progression from adenoma to adenocarcinoma.

Rodent models of lung carcinogenesis are effective tools for investigating the underlying mechanisms of human NSCLC development and for the detection of chemopreventive agents, as the morphologies, histogenesis and molecular characteristics of induced primary rodent lung tumors are similar to those of humans. The A/J mouse, which demonstrates increased spontaneous lung tumor development in the females than in the males, was selected for the present study. In addition, the female A/J mouse is particularly sensitive to lung carcinogenesis induced by 4-(methylnitrosamino)-1-(3-pyridyl)-1-butanone (NNK), a tobacco-specific nitrosamine component of tobacco smoke (31). *KRAS* mutations are frequently observed in this type of rodent lung carcinogenesis model, which was also noted in a case of human NSCLC (32).

In the present study, the expression of female hormone receptors (ER and PR), as well as EGFR and PCNA, was examined in NNK-induced lung tumors in female A/J mice (a rodent lung carcinogenesis model) in order to investigate the possible associations between different factors in mouse lung tumors, including adenomas, carcinomas in adenomas and adenocarcinomas.

## Materials and methods

### Chemicals

NNK was purchased from Toronto Research Chemicals Inc. (Toronto, Canada).

### Animals

Female A/J mice (age, 5 weeks), purchased from Shizuoka Laboratory Animal Center (Shizuoka, Japan) were maintained in the Division of Animal Experiments, Life Science Research Center, Kagawa University (Kagawa, Japan), in accordance with the Institutional Regulations for Animal Experiments. These regulations include the best considerations for animal welfare and good practice of animal handling, contributing to the replacement, refinement and reduction of animal testing. The protocol of the experiment was approved by the Animal Care and use Committee for Kagawa University. The animals were housed in polycarbonate cages with re-used paper chips (EchoChip^®^, CL-4163; CLEA Japan, Inc., Tokyo, Japan) for bedding, and given free access to drinking water and a basal diet (Oriental MF; Oriental Yeast Co., Ltd., Tokyo, Japan). The conditions were controlled as follows: Humidity, 60±10%; lighting, 12-h light/dark cycle; and temperature, 24±2°C. The experiments commenced following a 2-week acclimatization period.

### Experimental design and tissue preparation

Details of the original experiment were published by Takeuchi *et al* (33) and the tissue samples from one group in the experiment were used in the present study. Briefly, at the beginning of the current experiment, 20 mice (age, 7 weeks) were each administered with a single dose of NNK (2 mg/0.1 ml saline) via intraperitoneal injection. The mice did not receive any further treatment. The experiment was terminated at 52 weeks, when all of the surviving mice were euthanized under deep anesthesia, and their lungs were excised and weighed. The final weights of the lungs were calculated by subtracting the weights of the trachea and heart. The lungs were subsequently infused with 10% neutral-buffered formalin (Wako Pure Chemical Industries, Ltd., Osaka, Japan) and inspected grossly. In addition, the macroscopically detected lung nodules were counted under a stereomicroscope (Olympus SZ microscope; Olympus, Tokyo, Japan) and were routinely processed for embedding in paraffin. Histopathological examination with hematoxylin and eosin (H&E) was conducted by staining each lung lobe section. Proliferative lesions of the lung were diagnosed as hyperplasia, adenoma or adenocarcinoma according to the International Harmonization of Nomenclature and Diagnostic Criteria (INHAND) (34). Due to differences in terminology used in INHAND, ‘hyperplasia, bronchiolo-alveolar’, ‘adenoma, bronchiolo-alveolar’ and ‘adenocarcinoma, bronchiolo-alveolar’ were abbreviated to hyperplasia, adenoma and adenocarcinoma, respectively, in the present study.

In the H&E-stained slides, the number of lung proliferative lesions was counted and the major axis size of the lung tumors was measured under an Olympus BX51 microscope with an Olympus DP 70 digital camera, using an Olympus DP controller and the Olympus DP manager software (all Olympus).

To avoid errors and repeat counting, lung maps were prepared by transcribing all of the H&E-stained slides onto paper, and the tumor locations and histopathologic types were marked on each lung map during the microscopic investigation.

### Immunohistochemical analysis

The lung tissues were immunostained for EGFR, ER, PR and PCNA using the avidin–biotin complex method (Ventana Medical Systems; Oro Valley, AZ, USA); all of the processes, from deparaffinization to counterstaining with hematoxylin, were performed automatically using the Ventana Discovery™ staining system (Ventana Medical Systems). The primary rabbit anti-human EGFR polyclonal antibody, EGFR(1005), sc-03; anti-human ERα rabbit polyclonal antibody, ERα(H-184), sc-7207; and anti-human PR rabbit polyclonal antibody and PR(CR-19), sc-538 were purchased from Santa Cruz Biotechnology, Inc. (Santa Cruz, CA, USA) and were all used at a dilution of 1:50. Anti-human PCNA goat polyclonal antibody, PCNA(c-20), sc-9857 (Santa Cruz Biotechnology, Inc.) was used at a dilution of 1:400. The secondary antibodies used were biotinylated goat anti-rabbit IgG (BA-1000; Vector Laboratories, Inc., Burlingame, CA, USA) and biotinylated rabbit anti-goat IgG (BA-5000, Vector Laboratories, Inc.). The sections were incubated with the primary antibody (antibodies for ER, PR, and EGFR diluted at 1:50 and diluted at 1:400 for PCNA) for 12 h at 4°C and were incubated with the secondary antibody for 30 min.

The labeling indices were carefully assessed, to identify the cellular-positive areas (cellular membrane-, cytoplasm- or nucleus-positivity for each antibody in the tumor cells) whilst avoiding duplicate counting of the same tumor cells. Cells were considered to be positive when ER, PR, and PCNA staining was observed in the nuclei and when EGFR staining was noted on the cellular membranes.

The protocol for counting the labeling indices involved the following five steps to ensure accuracy: i) Drawing outlines of the histological H&E-stained slides of each lung tissue macroscopically on paper, termed lung maps; ii) marking the tumor locations and writing the histopathologic type on the lung map; iii) randomly selecting typical areas of each lung tumor, using an Olympus BX51 microscope equipped with an Olympus UPlanSApo 20x/0.75 objective lens (Olympus), and obtaining at least three images per single lung tumor without overlap using an Olympus DP 70 (Olympus) and Olympus DP controller and manager software; iv) printing all color images on A4 paper (210×297 mm) with a laser color printer, allowing counts of >1,000 cells per tumor lesion from three color copied sheets; v) counting the positive cells and putting a red tick mark on the first count and subsequently counting the negative cells with pencil marks on the second count.

### Statistical analysis

Correlations between the expression levels of EGFR, ER, PR, PCNA and the size of lung proliferative lesions were analyzed by Spearman’s rank test. P<0.05 was considered to indicate a statistically significant difference. Multiple comparisons between the labeling indices and the tumor size for the three histopathological types of lung tumors were conducted using the Tukey-Kramer test.

## Results

### Selection of lung tumor samples

Initially, there were 20 mice included in the study, however, one succumbed during the experiment and was not included in the effective numbers. Lung proliferative lesions, hyperplasia, adenoma and adenocarcinoma were diagnosed according to the criteria in the INHAND (34). Certain lung adenocarcinomas were identified within adenomas, therefore, these lesions were diagnosed as carcinomas in adenomas. All 19 animals exhibited adenomas (incidence, 100% and multiplicity, 7.79±3.44/mouse) and the total malignant tumors (a combination of lung adenocarcinomas and carcinoma in adenoma) were counted in 18/19 mice (incidence, 94.7% and multiplicity, 4.68±3.42/mouse). The carcinomas in adenomas accounted for 61.8% of the total malignant tumors and the remaining 38.2% were adenocarcinomas.

### Tumor characteristics

The investigated lung tumors, which could be observed in the H&E-stained slides as well as in all of the immunohistochemically-stained slides, were selected for evaluation. Samples were insufficient for staining in two mice due to their size, therefore, out of the remaining 17 mice, seven adenomas, seven carcinomas in adenomas and 17 carcinomas were selected. Only the carcinoma components were analyzed from the carcinomas in adenoma samples.

**Figure 1:**
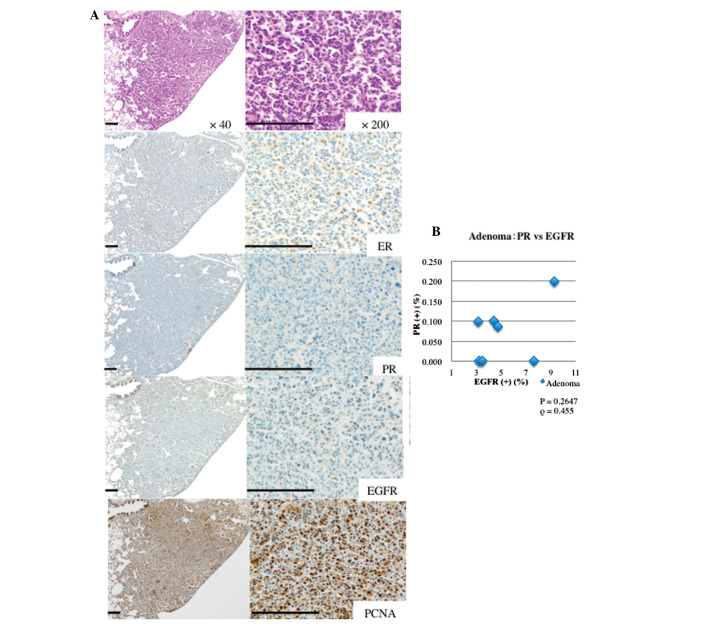
Histology and immunohistochemical findings for adenomas. (A) Histochemical and immunohistochemical findings of an adenoma (hematoxylin and eosin staining for ER, PR, EGFR and PCNA). (B) There was no correlation between the PR and EGFR labeling indices in adenomas. ER, estrogen receptor; PR, progesterone receptor; EGFR, epidermal growth factor receptor; PCNA, proliferating cell nuclear antigen.

**Figure 2:**
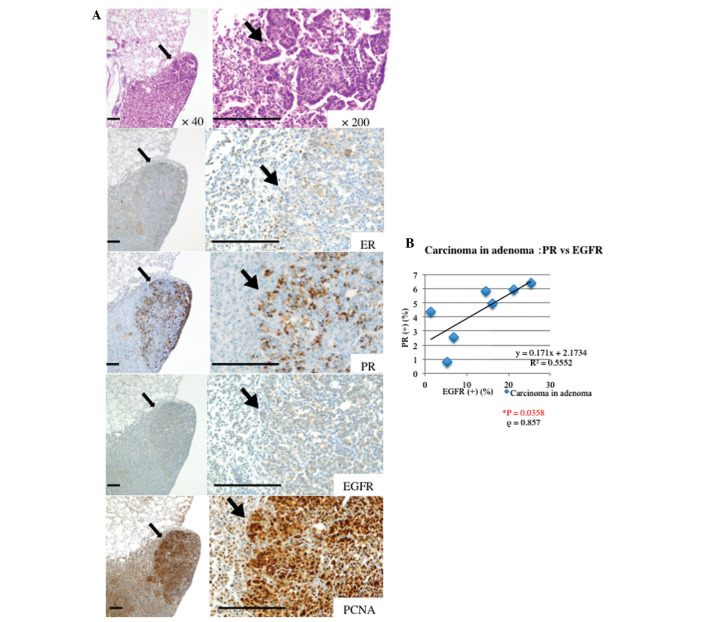
(A) Histochemical and immunohistochemical findings of carcinoma in adenoma (hematoxylin and eosin staining for ER, PR, EGFR and PCNA). Strong expression of PR, EGFR and PCNA was observed in the carcinoma component, although not in the peripheral adenoma component (black arrows). (B) A significant correlation (P<0.05) was observed between the PR and EGFR labeling indices of the carcinoma components of the carcinomas in adenomas. ER, estrogen receptor; PR, progesterone receptor; EGFR, epidermal growth factor receptor; PCNA, proliferating cell nuclear antigen.

**Figure 3:**
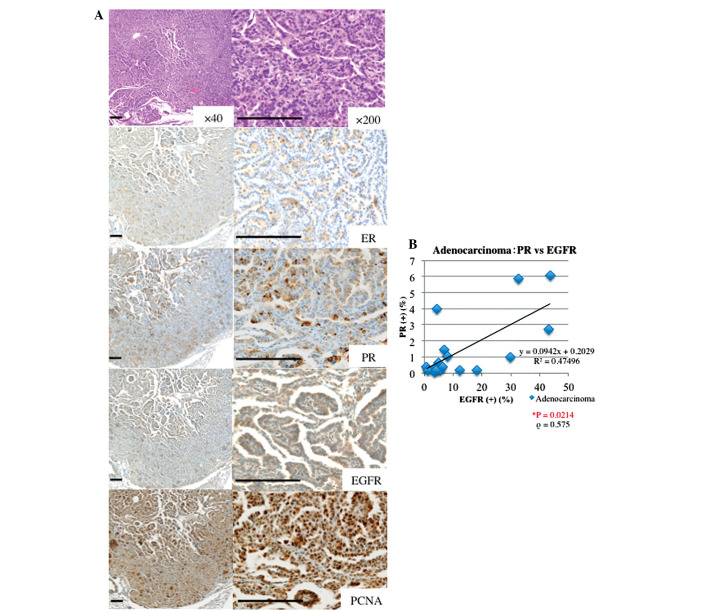
(A) Histology and immunohistochemical findings for adenocarcinomas (hematoxylin and eosin staining for ER, PR, EGFR and PCNA). (B) A significant correlation (P<0.05) between the PR and EGFR labeling indices was noted in the adenocarcinomas. ER, estrogen receptor; PR, progesterone receptor; EGFR, epidermal growth factor receptor; PCNA, proliferating cell nuclear antigen.

**Figure 4:**
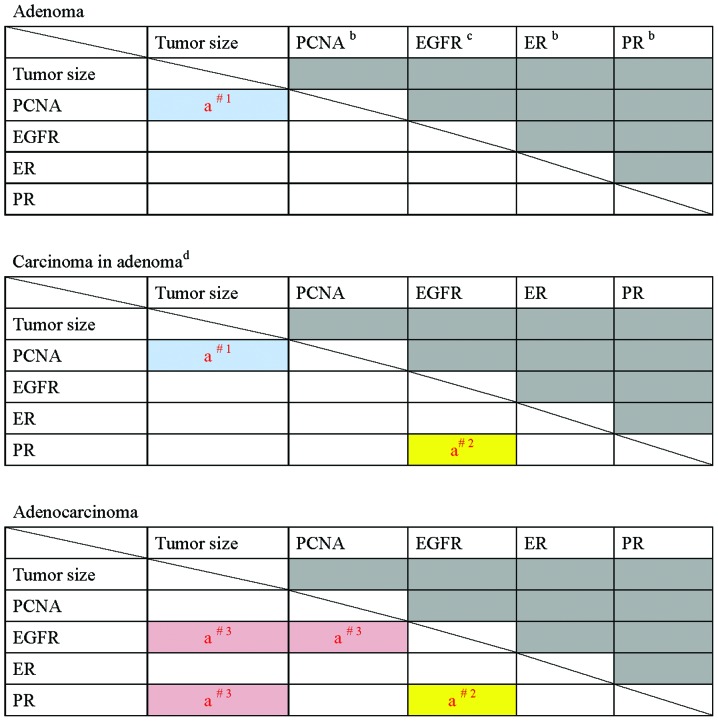
^a^Significant correlation (P<0.05 Spearman’s rank test); ^b^only cellular membrane expression was evaluated; ^c^only carcinoma components of the carcinoma in adenoma were evaluated; ^#1^significant correlation between the size of the tumor vs. the PCNA expression in the adenoma and the carcinoma in adenoma. Blue coloring signifies that the same tendency was observed in the adenoma and the carcinoma component of the carcinoma in adenoma; ^#2^significant correlation identified between PR vs. EGFR expression in the carcinoma in adenoma and the adenocarcinoma. Yellow coloring signifies that the same tendency was observed in the carcinoma component of the carcinoma in adenoma and the adenomcarcinoma; ^#3^in just adenocarcnima, significant correlation was identified between the size of the tumor vs. EGFR, the size of the tumor vs. PR and PCNA vs. EGFR expression. Red coloring signifies that the significant associations were only observerd in the adenocarcinoma. PCNA, proliferating cell nuclear antigen; EGFR, epidermal growth factor receptor; ER, estrogen receptor; PR, progesterone receptor.

**Figure 5:**
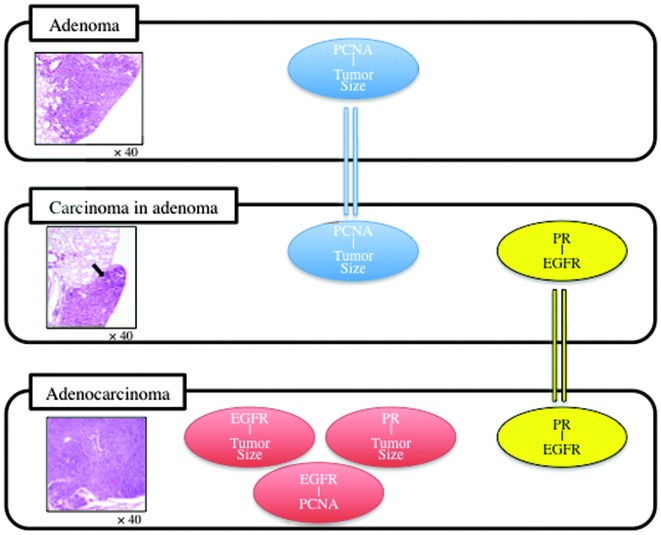
Schematic of the immunohistochemical findings, demonstrating the significant correlation between each histopathological type and the correlating factors within each of the three histopathological types. PCNA, proliferating cell nuclear antigen; PR, progesterone receptor; EGFR, epidermal growth factor receptor.

The histopathological and immunohistochemical findings from the three histopathological types of lung tumor nodules (adenomas, carcinomas in adenoma and adenocarcinomas) are summarized in Figs. 1–3. A strong expression of PR, EGFR and PCNA was observed in the carcinoma component of carcinomas in adenomas, in contrast to the peripheral adenoma component (Fig. 2). The major axis size of lung tumors on the H&E-stained slides was measured under a microscope. Data for the tumor size, and the labeling indices of the ER, PR, EGFR and PCNA-positive cells in each tumor type are summarized in Table I. Although, the adenomas tended to be larger than the other types of tumors, all of the immunohistochemical labeling indices (PCNA, ER, PR and EGFR) tended to be lower when compared with the carcinomas in the adenomas and adenocarcinoma groups. (Turkey-Kramer test: PCNA, P<0.01 and PR, P<0.001; Table I). Although PR-positive tumor cells were observed in the carcinomas in adenomas and in the adenocarcinomas, the adenomas were generally PR-negative. However, ER expression was similar in all of the histopathologic types. Using statistical analysis, correlation between five factors (the expression of EGFR in the cellular membranes, the expression of ER, PR, and PCNA in the nucleus and the size of the tumor [Fig. 4]) was detected using nonparametric correlation analysis (Spearman’s rank correlation) on 10 combined paired comparisons (_5_C_2_=10). The only statistically significant correlation between tumor size and PCNA expression was observed in the adenomas. Similarly, the carcinoma components of carcinomas in adenomas demonstrated significant correlations between tumor size and PCNA expression, as well as between EGFR and PR expression. No significant correlation between the size of the tumor and PCNA expression was observed in the adenocarcinomas. By contrast, significant correlations were identified between tumor size and EGFR expression and tumor size and PR expression. Furthermore, EGFR expression demonstrated a significant correlation with PR expression, as was also observed in the carcinoma components in carcinomas in adenomas. In addition, ER was not identified to be correlated with the other factors and no significant differences were noted between the three histopathological types. These findings are summarized in a schematic illustration in Fig. 5.

## Discussion

The present study demonstrated significant correlations between tumor size and the EGFR-positive indices, as well as between EGFR and PR expression in adenocarcinomas and the carcinoma components of carcinomas in adenomas. This indicates that PR and EGFR may have significant roles in tumor progression during NNK-induced mouse lung carcinogenesis. By contrast, ER expression was not identified to correlate with EGFR expression or tumor size; therefore, ER and PR may differ with regard to their influence on lung carcinogenesis.

A randomized prospective human study, as part of a multi-component clinical trial (the Coronary Drug Project) identified males who had previously suffered from a myocardial infarction. The aim was to evaluate the difference between the effects of administering 2.5 mg/day estrogen or a placebo; anticipating a reduction in future cardiac events. However, it was discontinued as a result of an increased incidence of lung cancer-associated mortality, which was observed in the estrogen treatment group (35). In addition, two randomized prospective trials proposed that hormone replacement therapy with estrogen plus progestins increased the incidence of and mortality from lung cancer in postmenopausal females (36,37). However, similar research with estrogen alone did not indicate increased lung cancer-associated mortality in females (38).

In mice, one study reported that the progression of spontaneous lung tumors was reduced with treatment using antiprogestin (Mifepristone; RU-486) (16).

Previously, mutations in codon 12 of the *KRAS* gene were detected in carcinogen-induced rodent lung tumor models (31), with similar incidences noted to those that were previously reported during an investigation of X-ray-induced rat lung tumors (39). Various molecular studies regarding human lung cancer indicate that the notable differences in *p53* and *KRAS* mutations are between the non*-*smokers and smokers. Overall, *KRAS* mutations have been identified to be more frequent in smokers compared with in non-smokers; although, those in codon 12 of the *KRAS* gene (G → T transversions and G → A transitions) are found in non-smokers and smokers (41). Conversely, although the frequency of mutation in the *p53* gene was similar in non-smokers when compared with smokers, the types and spectra of mutations were identified to be significantly different. Furthermore, C → T transitions are found only in non-smokers (40,41). The frequency of the EGFR mutation in human NSCLC is relatively high (26–40% in Asian patients and 2–12% in non-Asian patients). In one animal model, development of urethane-induced lung adenomas in male A/J mice was inhibited by an EGFR-tyrosine kinase (TK) domain inhibitor, despite a lack of any mutation in the EGFR-TK domain, which was proposed to be due to activated EGFR signaling (42). Thus, activation of the EGFR signaling pathway, caused by genetic mechanisms without somatic mutations of EGFR, may be involved in the growth of lung tumors in humans and animals. All the available data indicates that the variety and pathogenesis of NSCLC in never-smokers may be different compared with in smokers, with the involvement of additional factors considered to be significant.

In conclusion, this study demonstrated a significant correlations between tumor size and the EGFR-positive indices, as well as between EGFR and PR expression in adenocarcinomas and the carcinoma components of carcinomas in adenomas. This indicates that PR and EGFR may exhibit significant roles in tumor progression during NNK-induced mouse lung carcinogenesis. In order to study the participation of etiological factors in NSCLC progression, further studies are required to focus on the correlations between sex hormone functions and other growth factors in the sequential genesis of malignancies.

## Figures and Tables

**Table I:** Tumor size and immunohistochemical labeling indices for the positive cells in each tumor type^a^.

Variable	Adenoma	Carcinoma in adenoma^c^	Adenocarcinoma
Tumors, n	7	7	17
Tumor size, mm	1.9±0.4	0.6±0.3^d^	1.8±0.7
Labeling indices, %			
PCNA^b^	60.7±13.0	84.1±5.4^e^	71.8±12.6
EGFR	5.1±2.4	13.0±8.7	13.5±14.5
ER^b^	6.4±3.1	6.7±3.2	6.7±4.5
PR^b^	0.1±0.1	4.4±2.0^d^	1.5±2.0

a Values are expressed as the mean ± standard deviation;

b expressed as nuclear-positive;

c only the carcinoma components were evaluated;

d significantly different compared with adenomas (P<0.001; Turkey-Krammer test);

e significantly different compared with adenomas (P<0.01; Turkey-Krammer test).

PCNA, proliferating cell nuclear antigen; EGFR, epidermal growth factor receptor; ER, estrogen receptor; PR, progesterone receptor.
